# Attention spotlight in V1-based cortico-cortical interactions in human visual hierarchy

**DOI:** 10.1038/s41598-024-63817-y

**Published:** 2024-06-07

**Authors:** Yanyu Zhang, Xilin Zhang, Xincheng Lu, Nihong Chen

**Affiliations:** 1 Institute of Neuroscience, Center for Excellence in Brain Science and Intelligence Technology, Chinese Academy of Sciences, Shanghai, 200031 China; 2 Department of psychological and cognitive sciences, Tsinghua University, Beijing, China; 3 IDG/McGovern Institute for Brain Research at Tsinghua University, Beijing, China; 4 Key Laboratory of Brain, Cognition and Education Sciences, Ministry of Education, South China Normal University, Guangzhou, 510631 Guangdong China; 5 School of Psychology, Center for Studies of Psychological Application, and Guangdong Provincial Key Laboratory of Mental Health and Cognitive Science, South China Normal University, Guangzhou, 510631 Guangdong China

**Keywords:** Spatial attention, fMRI, Connectivity, DCM, V1, Visual cortex, Attention, Extrastriate cortex, Striate cortex

## Abstract

Attention is often viewed as a mental spotlight, which can be scaled like a zoom lens at specific spatial locations and features a center-surround gradient. Here, we demonstrate a neural signature of attention spotlight in signal transmission along the visual hierarchy. fMRI background connectivity analysis was performed between retinotopic V1 and downstream areas to characterize the spatial distribution of inter-areal interaction under two attentional states. We found that, compared to diffused attention, focal attention sharpened the spatial gradient in the strength of the background connectivity. Dynamic causal modeling analysis further revealed the effect of attention in both the feedback and feedforward connectivity between V1 and extrastriate cortex. In a context which induced a strong effect of crowding, the effect of attention in the background connectivity profile diminished. Our findings reveal a context-dependent attention prioritization in information transmission via modulating the recurrent processing across the early stages in human visual cortex.

## Introduction

Attention can be deployed upon a specific zone of visual field, enhancing the processing of stimuli at this region^1^. The selection of spatial attention has often been conceptualized with a spotlight metaphor. It has been suggested that the attention spotlight can be scaled like a zoom lens^2^, organized in gradient^3–5^, and may come with a suppressive surrounding^6,7^.

Regarding the communication of attention signals across visual processing stages, a long-standing hypothesis posits that feedback connectivity delivers top-down signals that influence the sensory representation in the early visual cortex. Feedback connections serve to amplify and focus activity of neurons in lower-order areas^8–11^. Attention-related interactions have been found in the visual hierarchy^12–14^. However, the spatial properties, i.e., the center-surround modulation profile, in cortico-cortical connectivity remain less clear. In particular, feedback activity has been shown to modulate the response amplitude and surround suppression in the primary visual cortex^8,15^, showing a similar effect of spatial attention on local activation in V1 and V4^16,17^. Such a modulation effect remains to be evidenced in visual-spatial tasks in inter-areal interaction.

Human magnetoencephalographic (MEG) studies have indicated that surround suppression is linked with a stronger recurrent activity modulation in the early visual cortex, occurring beyond the time window of the initial feedforward sweep^18,19^. In addition, attention-related enhancement has been found in the preparatory activity in the absence of visual stimulation^20–22^. These results also support a critical role of recurrent processing in modulating the attention-dependent signal in the visual hierarchy.

**Figure 1:**
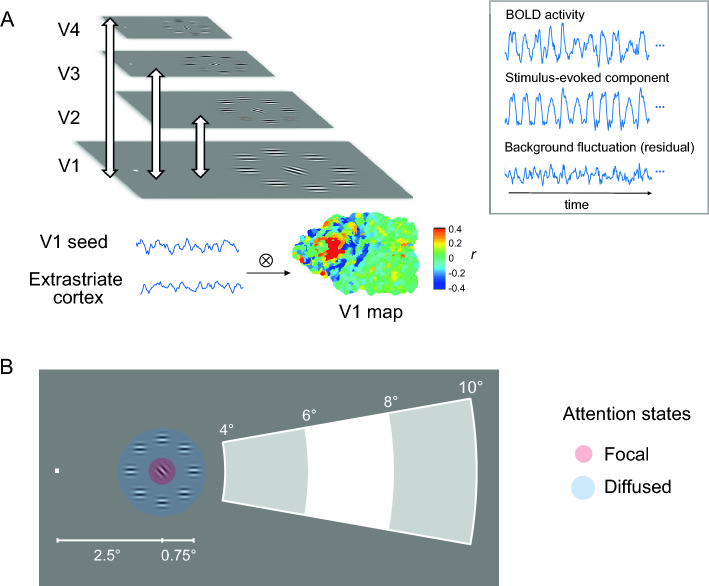
Schematic description of background connectivity analysis and experimental design. (**A**) Background connectivity analysis was performed on the residual BOLD signals between vertexwise V1 on the inflated surface and the ROIs responsive to the stimuli in V2–V4. Left panel: stimuli-evoked responses were estimated with FIR basis functions to capture the timing and shape of the hemodynamic response. Residuals from the evoked model were used for assessing background connectivity under each attention condition. Right panel: V1 map derived from the correlation analysis of residuals between vertexwise seed region in V1 and downstream visual areas. (**B**) Schematic description of stimuli and design. Subjects’ attention was either focused on the central element of the multi-item array, or deployed diffusely over the array. The spatial distribution of background connectivity pattern, binned according to the eccentricity in the non-stimulated peripheral visual fields, was compared between the two attention conditions.

To further characterize the spatial profile of attentional modulation in the inter-areal communication and its context dependency, we performed V1-based background connectivity and dynamic causal modeling based on fMRI BOLD signals, while subjects were performing tasks with focal or diffused attentional states over the visual field. We plotted the background connectivity map based on spatial coordinates of the visual field representations in relation to the attended focus. Our visual stimuli were orientation patches consisting of a target at 2.5° horizontal eccentricity and eight surrounding flankers (Fig. 1B). Experiment 1 examined the attention effect with iso-oriented flankers which induced a weak behavioral crowding effect. Experiment 2 examined the attention effect with heterogeneous flankers which induced a strong behavioral crowding effect. During the scan, subjects were asked to attend all the patches, or to attend only the target. We investigated how attention-induced bias in space is encoded via the interplay between V1 and extrastriate cortex at a fine spatial metric.

## Methods

### Subjects

A total of 12 subjects (5 female, 23–38 years old) were enrolled in the experiment. Eight participated in the first experiment, and eight participated in the second experiment. All subjects had normal or corrected-to-normal vision. They gave written, informed consent and the procedures were conducted in accordance with the procedures relevant guidelines and regulation and the protocols were approved by the human subject review committee of University of Southern California.

### Stimuli and apparatus

All the behavioral and fMRI experimental procedures are described according to Chen et al.^23^. A Gabor at an eccentricity of 2.5° to the right of fixation was surrounded by eight flanking Gabors (diameter: 0.625°; 0.75° from the central Gabor). In the first fMRI experiment, all the flankers had a horizontal orientation. In the second fMRI experiment, the orientation of each flanker was randomly assigned from 0°, 45°, 90° and 135°. Before the scan, subjects were asked to identify the orientation of the central Gabor with and without the flankers. Subjects were asked to maintain the central fixation and to make a four-alternative-forced-choice judgment of the target orientation with a button press. The next trial began 1000 ms after response. For each condition, the performance was averaged over 40 trials.

### fMRI procedures

BOLD signals responding to the multi-element stimuli were measured in block-design fMRI scans. Subjects maintained fixation while attending to the Gabor stimuli throughout the run. In a stimulus block, all Gabors in the array were counterphase flickering at 2 Hz with random pauses. The contrast of the central Gabor decreased at random time points. In the focal attention condition, subjects detected a reduction in the contrast of the central Gabor in the array. In the diffused attention condition, subjects detected a pause of flickering of the whole Gabor array. The task order was counterbalanced across subjects. For each attention condition, 8 runs were measured. Each stimulus block lasted for 12 s and was interleaved with 12 s fixation blocks (see Chen et al.^23^ for details).

### MRI data acquisition and preprocessing

The present study analyzed data from Chen et al.^23^. MRI data were collected using a 3T Prisma scanner with a 32-channel phase-array coil (Siemens). BOLD signals were measured at a resolution of 3 × 3 × 3 mm^3^ with a multiband EPI sequence (TE: 35 ms; TR: 1 s; FOV: 192 × 192 mm^2^; matrix: 64 × 64; flip angle: 63°; slice thickness: 3 mm; gap: 0 mm; number of slices: 42; slice orientation: axial). A high-resolution 3D structural dataset (MPRAGE; 0.8 × 0.8 × 0.8 mm^3^ resolution) was collected. MRI data analyses were performed using Freesurfer (version 5.3) and FSL (version 4.1, FMRIB’s Software Library). The anatomical volume was processed using Freesurfer to reconstruct the inflated cortical surface for each subject. The functional volumes were preprocessed using FSL, including motion-correction and high-pass temporal filtering. All functional datasets were individually registered into 3D space using the subjects’ individual high-resolution anatomical images.

### Regions of interest

Retinotopic mapping of visual areas V1, V2, V3, and V4 was performed using a standard phase-encoded method^24,25^, in which subjects viewed a rotating wedge and an expanding ring that created traveling waves of neural activity in visual cortex. Two independent localizer runs, identical to the runs in the main experiment, were used to identify voxels in each area that showed a stronger response to stimulus conditions than fixation (p < 0.01). In V1, in addition to the stimulus-evoked region, peripheral ROIs were defined on the cortical surface based on the polar atlas and eccentricity atlas from the 7T retinotopic dataset of Human Connectome Project^26^. Vertices in the contralateral hemisphere with a polar angular between ± 10° from the horizontal meridian were binned into three ROIs: 4–6°, 6–8°, 8–10° according to their eccentricity (Fig. 1B).

### Background connectivity

Background connectivity was measured between residual BOLD signals from stimulus-evoked regions in V2–V4 and every voxel in V1 within the same hemisphere, following the previous V1-based retinotopic connectivity procedure^27^. The residuals were derived after removing confounding variables and stimulus-evoked responses^28,29^. Signals from white matter and ventricle, along with six motion parameters, were regressed out of the preprocessed BOLD signal timeseries in a GLM for each run. Then, we estimated stimulus-evoked responses with finite impulse response (FIR) basis functions that captured the mean evoked response across blocks. Each block and subsequent fixation period were modeled by 24 delta functions, one for each TR. Residuals from the evoked model were used for assessing functional connectivity under each attentional state, which was independent of the correlation attributable to stimulus-evoked responses.

### Dynamic causal modeling (DCM)

The DCM analysis^30,31^ was performed to examine the attentional effect on the effective connectivity between the V1 area representing the target stimuli and the V1 area representing the peripheral blank visual field (referred to as V1_S and V1_NS, respectively), and the stimulated areas in V2, V3, and V4. V1_NS was defined at an eccentricity of 6–8° as it demonstrated a suppression effect in focal attention versus diffused attention condition in background connectivity analysis. The first set of parameters were extrinsic inputs into the stimulated region of V1. fMRI timeseries from all the runs under both attention states were concatenated and modeled with a GLM procedure, with the regressor for all the stimulus blocks (i.e., the extrinsic input to V1_S). The second set of parameters were intrinsic connectivities among the four modeled nodes, with bidirectional intrinsic connections. The third set of parameters were bilinear parameters encoding the modulation of focal attention relative to the diffused attention state on the specified intrinsic connections. We examined three models which differed in the modulatory sites, hypothesizing that focal attention functions on the feedforward, feedback, or recurrent connectivities. Assuming heterogeneity across subjects in terms of the modulatory effect on intrinsic connectivity^32^, a random effects (RFX) analysis was used to compare the models. The models were compared by computing the exceedance probability of each model, i.e., the probability to which a given model is more likely than the other two models to have generated data from a randomly selected subject. In the best model, we examined changes in the modulatory effects.

### Estimating stimulus field

We adopted the method in Herrmann et al.^33^ to quantify the center and the size of stimulus field. The spread of the spatial distribution of BOLD response across the eccentricities was considered as a probability distribution and was quantified using the following equations.

$$\text{E}\left(\text{x}\right)= \sum {\text{x}}_{\text{i}}\text{ p}\left({\text{x}}_{\text{i}}\right)$$

E(x) represented the center of the attention field and had units of degrees of visual angle. x_i_ is the eccentricity and p(x_i_) is the response at this eccentricity. Negative values were truncated, and responses were normalized such that they summed to 1.

$$\text{Sd}\left(\text{x}\right)= \sqrt{\sum {\left({\text{x}}_{\text{i}}-\text{E}\left(\text{x}\right)\right)}^{2}\text{ p}\left({\text{x}}_{\text{i}}\right)}$$

The size of the attention field was defined as twice the SD in the visual field. This analysis was applied for both attentional conditions in each subject (Fig. 6).

## Results

### Attention spotlight in V1-based inter-areal connectivity

We performed background connectivity analysis to measure the neural coupling between vertex-wise V1 and the extrastriate cortex, measured as *Pearson* correlation coefficients between the residual timeseries of all the individual data points on the V1 surface and the residual timeseries from each stimuli-responsive region in areas V2–V4. In the focal attention condition, the connectivity on the flattened V1 map shows a trend of enhanced neural coupling at the stimulated area, while its neighboring region shows a trend of reduced neural coupling (Fig. 1A).

**Figure 2:**
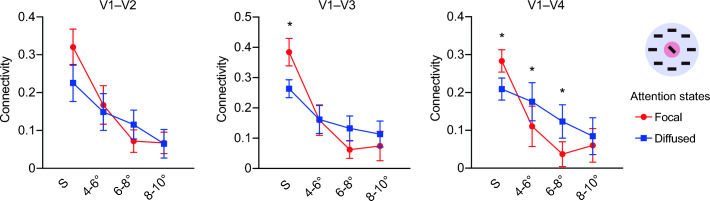
Background connectivity under focal and diffused attention conditions. Error bars denote ± 1 SEM across subjects. *p < 0.05.

At the V1 area representing the visual field further away from the focus of attention, the inter-areal connectivity strength decreased in the focal attention state compared to the diffused attention state (Fig. 2). A repeated-measures ANOVA shows a significant main effect of visual field in the connections between V1 and downstream areas (all F(3, 21) > 11.14, p < 0.01). Importantly, a significant interaction between visual field and attention was observed in V1–V3 connectivity (F(3, 21) = 4.90, p < 0.01), as well as in V1–V4 connectivity (F(3, 21) = 7.67, p < 0.01). In V1–V4 connectivity, a clear pattern of center enhancement and surround suppression was identified. Focal attention enhanced the neural coupling between the stimulated V1 area and V4 (paired t-test, t(7) = 2.92, p < 0.05), and suppressed the coupling between the non-stimulated areas at peripheral eccentricity bins of 4–6° and 6–8° (paired t-test, both t(7) > 2.54, p < 0.05). The same connectivity pattern was preserved before and after removing the stimulus-evoked components (Supplementary Fig. S1).

### Effect of focal attention in both feedforward and feedback processing

**Figure 3:**
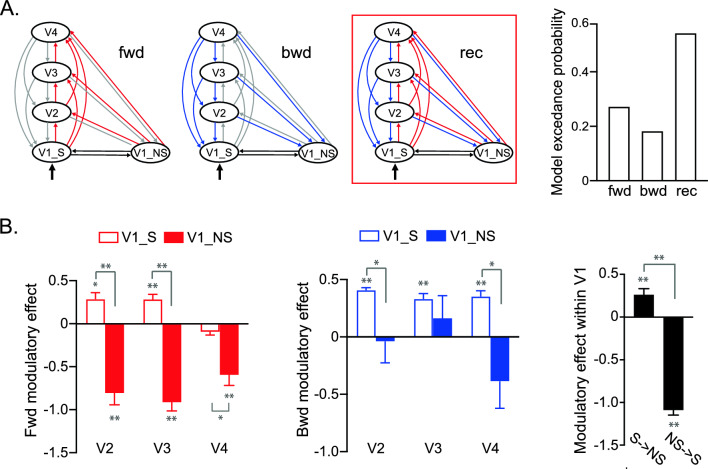
DCM results. (**A**) Candidate models and Bayesian model selection results. Models differ in their modulatory sites, hypothesizing that the effect of focused versus diffused attention on the feedforward (red), feedback (blue), or recurrent connections (red and blue), respectively. The exceedance probability for each model was computed, i.e., the probability to which a given model is more likely than the other two models to have generated data from a randomly selected subject. (**B**) Parameter estimates of the modulation effect in the best model, on the connections between the stimulated V1 area (V1_S), the non-stimulated V1 area (V1_NS), and V2–V4. Error bars denote ± 1 SEM across subjects. *p < 0.05; **p < 0.01; FDR corrected.

Using DCM, we tested three models: feedforward, feedback, and a recurrent model, each differing in the site of attentional modulation (focused vs. diffused; Fig. 3A). Bayesian model selection analysis shows that the recurrent model, which incorporates attentional modulation on both feedforward and feedback connections, has the highest exceedance probability. Figure 3B demonstrated a contrast in the modulatory effects between the connectivity with the V1 area representing the stimuli (V1_S) and the area representing the peripheral non-stimulated visual field (V1_NS). Generally, the contrast was manifested as enhancement in the connection with V1_S, and suppression in the connection with V1_NS. For the feedforward connections, focal attention amplified the difference in the connectivity strength between V1_S and V1_NS to downstream areas. A repeated-measures ANOVA with visual field and cortical area as two factors revealed a main effect of visual field (all F(1,7) = 439.42, p < 0.01; paired t-test, all t(7) > 3.28, p < 0.05). In the feedback connection, a similar pattern was observed in the modulatory effects between V1_S and V1_NS (main effect of visual field: F(1,7) = 185.01, p < 0.01), with feedback connections from V2 and V4 showing a significant modulation contrast (paired t-test, both t(7) > 2.65, p < 0.05). For local connectivity within V1, focal attention also magnified the modulation contrast (paired t-test, t(7) = 13.61, p < 0.01). These results suggest that spatial attention reweighted the recurrent connections between V1 and the extrastriate cortex, as well as the local connections within V1.

### Absent effect of focal attention in a context with a strong crowding effect

Outside the scanner, we compared subjects’ identification accuracy between the target-plus-flanker condition and the target-only condition. In the configuration with iso-oriented flankers, the presence of iso-oriented flankers resulted in a weak behavioral crowding effect, with a 3% reduction in target identification accuracy (t(7) = 2.31, p = 0.06). To investigate the contextual dependency, we tested the condition with flankers of heterogeneous orientations. A significant crowding effect was observed: the presence of flankers reduced the identification accuracy by 20% (t(7) = 4.25, p < 0.01).

**Figure 4:**
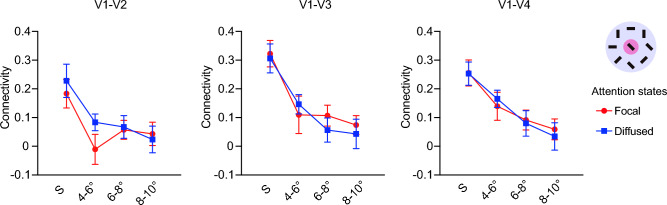
Background connectivity results in the display with heterogeneous flankers. Error bars denote ± 1 SEM across subjects.

For the condition with heterogeneous orientations, little attention effect was observed on the background connectivity (Fig. 4). A repeated-measures ANOVA with factors of eccentricity and attention showed a significant main effect of eccentricity (all F(3, 21) > 4.73, p < 0.05). However, no significant effect was found for main effect of attention (all F(1, 7) < 3.30, p > 0.05), or the interaction effect (all F(3, 21) < 2.19, p > 0.05).

**Figure 5:**
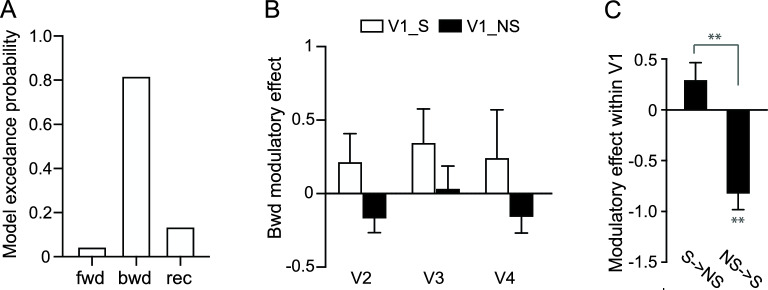
DCM results in the display with heterogeneous flankers. (**A**) Exceedance probability for each candidate model. (**B, C**) Parameter estimates for the modulation effect in the best model. Error bars denote ± 1 SEM across subjects. *p < 0.05; **p < 0.01; FDR corrected.

In the heterogeneous context, DCM model comparison shows that the feedback model has the highest exceedance probability (Fig. 5A). We further examined the modulatory effect on the feedback connectivity (Fig. 5B) and the connectivity within V1 (Fig. 5C). There was no significant difference between V1_S and V1_NS in the background connectivity between visual processing stages (main effect of visual field: F(1,7) = 19.8, p < 0.01; post-hoc paired t-test, all t(7) < 1.77, p > 0.05). Focal attention only enhanced the contrast between the connectivity within V1 (paired t-test, t(7) = 4.50, p < 0.01), with a significant suppression in the connectivity from the peripheral non-stimulated V1 to the stimulated V1 area (one-sample t-test, t(7) = 5.28, p < 0.01).

**Figure 6:**
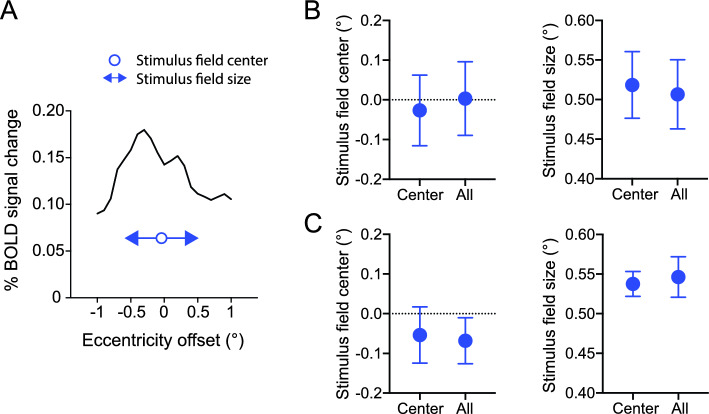
Stimulus field measurements. (**A**) Illustration of measurements on the distribution of BOLD response in V1. (**B**, **C**) Stimulus field center and stimulus field size in the context of weak crowding (**B**) and strong crowding (**C**). Error bar denotes 1 SEM across subjects.

A potential concern is that the eye movements might account for the attention effect reported above. If this is the case, different eye movements should result in different retinotopic distributions of BOLD response to the stimulus array. Based on the BOLD response distributions in V1, we quantified the center and the spread size of the stimulus field for each attentional condition (Fig. 6). In both the context with a weak crowding effect (Fig. 6B) and the context with a strong crowding effect (Fig. 6C), the measured centers of the stimulus field did not differ between the attention conditions (paired t-test, both t(7) < 1.00, p > 0.05), and were close to the center of the physical stimuli (one-sample t-test, all t(7) < 1.17, p > 0.05). Neither did the field size differ between the attention conditions (both t(7) < 1.91, p > 0.05). These results suggest that eye movement is unlikely to account for our findings.

## Discussion

Using fMRI and V1-based connectivity analysis, we investigated how spatial attention modulates information transmission in the visual hierarchy. Our study provides the following findings: (1) Focal attention, compared to diffused attention, sharpened the spatial gradient in V1-based neural coupling with extrastriate visual cortex; (2) Attention-dependent facilitation-inhibition in spatial gradient was identified in both the feedforward and the feedback connectivity; (3) In the context with strong crowding effect, no significant attentional modulation was found in the recurrent processing. These findings suggest focal attention facilitates information transmission via modulating the recurrent processing across the early stages in human visual cortex.

Macaque neurophysiological studies have long established that attention plays a crucial role in visual processing by enhancing the neural response in the visual area representing the target^34,35^. Similarly, human fMRI and electrophysiological studies have reported attention-dependent modulation in neural activation in the retinotopic visual areas^36–41^. Using a retinotopic mapping paradigm of spatial attention, a neural correlate of the attentional spotlight has been reported in human V1^41^. Moreover, a suppressive zone in the vicinity of the attended field, described as the ‘penumbra’ of attention spotlight, was identified^38^, and was found to extend to distant fields apart from the attended location^42^.

Using V1-based background correlation, our findings reveal a neural substrate of selective attention. In the context with weak crowding effect, we detected attention-dependent sharpening of the spatial gradient in inter-areal connectivity. The spatial distribution of this attentional effect aligns with a zoom-lens model^7,43,44^, showing that focal attention facilitated connectivity at the stimulus field and suppressed connectivity in adjacent areas, compared to the diffused attention state. The DCM results further elucidate the directionality of attention-dependent modulation. In both feedforward and feedback connectivity, the center-surround modulation was observed. Our results suggest that a narrowed attention zone can be achieved by optimizing recurrent connections in the visual hierarchy.

The role of recurrent connections in attention echoes the framework of predictive coding^45–48^, where perception is achieved via an interplay between top-down expectation and sensory-driven processes. According to an expanded framework of the predictive coding model, attention acts as a selective sampling of sensory information, prioritizing the target with high precision relative to predictions^49^. These predictions then cascade through the visual hierarchy^48,50–52^. Consistent with this perspective, we observed an increasing trend of attention-dependent sharpening along the visual hierarchy, with V4 establishing the strongest effect of surround suppression.

Notably, the recurrent modulation of attention only existed in the context of a weak crowding effect. In the presence of heterogeneous context, no significant difference in connectivity was found between focused and diffused attention. Recently, we have reported an overall enhancement in the time-lagged background connectivity from the pulvinar to V1, induced by focused attention^27^. This enhancement suggests the initiation of attention under conditions with strong crowding. However, the background connectivity was indistinguishable between attention states at the time lag of zero, aligning with the lack of effects in V1 connectivity observed in this study. This suggests that while attention can be initiated in the condition of strong crowding, its impact on sustained inter-areal connectivity may reflect the success of selective attention allocation.

In both contexts, DCM results showed the effect of focal attention on within-V1 interactions between target and flankers, which can be mediated via long-range horizontal projections within visual areas^53–55^. This local processing was in line with the notion that local gradient of sufficient strength is a necessary pre-requisite for segregation and creating a bottom-up saliency map^56–59^. Nevertheless, the local processing in V1 may not be sufficient for a successful implementation of focused attention. The behavioral relevance of inter-areal interaction in the visual hierarchy, aided by local processing in V1, may underpin the target selection process in crowding scenes^8,60–62^.

Recent advances in functional and effective connectivity analyses have enabled fMRI investigations in the interplay between the visual areas that go beyond local BOLD signal changes. The background connectivity analysis was based on the residual signals after the removal of stimulus-evoked responses. Such fluctuation in background neural activity is believed to reflect dynamic changes of the intrinsic state of the visual cortex^63–65^. This type of inter-areal connectivity has been shown to be modulated by attention in the visual hierarchy^28,29,61,66^. The long-range synchrony may provide a common temporal reference, like the binding-by-synchronization hypothesis, which facilitates communication between cortical areas^67–69^.

In sum, our findings characterize the spatial profile of V1-based inter-areal connectivity in focal and diffused attentional states. The interaction within the early-to-mid visual hierarchy shed light on the intricate attention network, revealing critical roles of feedforward and feedback inter-areal interplay, and local interactions in target selection in crowding. It should be noted that our conclusions regarding the attention effect were drawn from comparisons between focused and diffused attention conditions. The effect of enhancement and suppression relative to a no-attention baseline on connectivity remained to be examined in future studies. With V1 serving as an innate, high-resolution reference, retinotopic connectivity analysis sets a ubiquitous platform for studying topographic communication in the brain network^70^. Future fMRI research utilizing the background connectivity approach will also extend our understanding of the visuo-frontal interactions beyond the visual cortex and how the thalamo-cortical processing orchestrates with cortico-cortical processing in the attention network.

### Supplementary Information

Supplementary Figure S1.

## Data Availability

The data that support the findings of this study are available from the corresponding author upon reasonable request.
