# Supplementary material for: Attention spotlight in V1-based cortico-cortical interactions in human visual hierarchy
Source: Sci Rep. 2024 Jun 7;14:13140. doi: 10.1038/s41598-024-63817-y (PMC11161588; doi:10.1038/s41598-024-63817-y)
Supplement: Supplementary file 1 — Supplementary Figure S1. [file 41598_2024_63817_MOESM1_ESM.docx]

**Supplementary Materials**

Supplementary Figure S1. Removing the stimulus-evoked component preserves the connectivity pattern

As the background connectivity between V1 and V4 showed a suppressive effect of focal attention at the non-stimulated area with an eccentricity ranging from 6° to 8°, we further checked its connectivity pattern before the removal of stimulus-driven responses. The interaction between the ROIs in V1 (S/NS) and the attention state (Focal/Diffused) was significant before the removal of stimulus-evoked response (F(1,7) = 18.85, p < 0.01; Supplementary Fig. S1A), and after the removal of stimulus-evoked response (F(1,7) = 39.69, p < 0.01; Supplementary Fig. S1B).
